# Black and minority ethnic group involvement in health and social care research: A systematic review

**DOI:** 10.1111/hex.12597

**Published:** 2017-08-15

**Authors:** Shoba Dawson, Stephen M. Campbell, Sally J. Giles, Rebecca L. Morris, Sudeh Cheraghi‐Sohi

**Affiliations:** ^1^ NIHR Greater Manchester Primary Care Patient Safety Translational Research Centre (Greater Manchester PSTRC) Faculty of Biology, Medicine and Health Division of Population Health, Health Services Research and Primary Care School of Health Sciences The University of Manchester Manchester UK

**Keywords:** black and minority ethnic group, health and social care, patient and public involvement, research

## Abstract

**Background:**

Patient and public involvement (PPI) in research is growing internationally, but little is known about black and minority ethnic (BME) involvement and the factors influencing their involvement in health and social care research.

**Objectives:**

To characterize and critique the empirical literature on BME‐PPI involvement in health and social care research.

**Search strategy:**

Systematic searches of six electronic bibliographic databases were undertaken, utilizing both MeSH and free‐text terms to identify international empirical literature published between 1990 and 2016.

**Inclusion criteria:**

All study designs that report primary data that involved BME groups in health or social care research. Screening was conducted by two reviewers.

**Data extraction and synthesis:**

Data extraction and quality appraisal were performed independently. Data extraction focused on the *level(s*) of PPI involvement and *where* PPI activity occurred in the research cycle. Studies were quality‐assessed using the guidelines for measuring the quality and impact of user involvement in research. Data were analysed using a narrative approach.

**Main results:**

Forty‐five studies were included with the majority undertaken in the USA focusing on African Americans and indigenous populations. Involvement most commonly occurred during the research design phase and least in data analysis and interpretation.

**Conclusion:**

This is the first systematic review investigating BME involvement in health and social care research internationally. While there is a widespread support for BME involvement, this is limited to particular phases of the research and particular ethnic subgroups. There is a need to understand factors that influence BME involvement in all parts of the research cycle.

## 1 INTRODUCTION

Patient and public involvement (PPI) in research has been defined as “research being carried out ‘with’ or ‘by’ members of the public rather than ‘to’, ‘about’ or ‘for’ them”.^1^ Involving patients and public in research has become central to the health research policy context, both in the UK and internationally.^2^, ^3^, ^4^ This is reflected in the proliferation of official guidance on principles of best practice in PPI in countries such as UK,^1^ Canada^3^ and Australia.^5^ The growth of PPI has been underpinned by suggestions that actively involving service users in all aspects of the research process has the potential to improve research quality, relevance, impact and integrity as users can provide unique and relevant perspectives.^6^, ^7^ There is also a moral or human rights aspect to involving those who are the recipients of care and responsible for financing these services, either in a direct (fee for service) or indirect (eg, via taxation funded systems) manner.^8^ In the UK, interest in PPI within the National Health Service stems from demands of the public for a greater voice and involvement in making decisions regarding their services and politicians’ demand for greater efficiency, service quality and effective use of public funds that reflect the influence of the New Public Management approach to health services.^9^

Terminology within this area is varied and lack consistency, as researchers tend to use participation^1^ engagement^2^ and involvement interchangeably.^10^ This study will focus on involvement of people from Black and Minority Ethnic (BME) backgrounds, as existing systematic reviews in the area of PPI and health and social services have assessed the impact of PPI on health and social care research^4^ and UK health‐care services more widely.^11^, ^12^ The reviews offered evidence that involvement can have positive impact on research in terms of enhanced research quality, relevance and ensuring appropriateness^4^, ^11^, ^12^ as well as identifying a smaller evidence base of challenges to PPI^4^ such as potential tensions between researchers and PPI contributors^3^ due to different perspectives; results may not be perceived as important and concern that PPI contributors may disseminate the results before publishing in academic journals. Moreover, these studies^10^, ^11^, ^13^ found that a key limitation to PPI evidence base was poor quality of reporting impact, with a small number of studies defining PPI, little theoretical conceptualization, lack of robust measures of impact and lack of detailed account of descriptive evidence.

Other reviews on the topic have focused on how to *identify* and engage patients in health services research, as well as benefits and barriers to patient engagement.^14^ Despite the widespread promotion and inclusion of PPI in the last ten years, some have suggested that involvement is limited in scope as to *whom* is involved, with PPI activity not mirroring the diversity of the population.^10^, ^15^, ^16^ For example, studies have reported either a difficulty in involving or a lack of involvement of diverse groups (eg, BME groups) in health and social care research.^17^, ^18^ In addition, people from BME groups often experience inequalities in care,^19^, ^20^, ^21^ may also have specific health and social care needs and/or views on service design/provision, which, if not taken into account during the research or service design phase, could mean that the end product/services(s) might not necessarily reflect their specific needs and therefore might not be used/acceptable and therefore effective. In order to address this, it is not only important but also necessary to involve members of BME communities in a PPI capacity so that health and social care research is more relevant to them and produces appropriate research and health outcomes.

Traditionally, in Western literature, the term BME, Black, Asian and Minority Ethnic (BAME) Groups or “ethnic minority” has been typically used to refer to minority populations characterized by their non‐White origin.^22^, ^23^ However, the definition of minority ethnic groups varies and is naturally defined and described differently in different countries. The label of BME is therefore somewhat of a blunt instrument, which attempts to provide comprehensive coverage for what are highly nuanced, locally defined populations which may likely have differing health and social care needs. It is difficult therefore to have a holistic definition for ethnic minority groups that can be applied globally and which acknowledges this complexity. However, the BME label is seemingly most prominently used and recognized within the health and social care literature, the topic of this research and therefore will be utilized in this study. Furthermore, this study will utilize from the included studies, the authors’ reported definitions of what constitutes a BME population in their individual studies.

In summary, although the lack of BME involvement in health and social care research is clearly identified as an issue from the existing PPI literature in the area, no study to date has provided a systematic review of PPI for people from BME backgrounds in health and social care research. This study intends to fill this gap in our current knowledge.

This study therefore aims to review the literature in order to:

1. Identify, characterize and synthesize the literature on the involvement of people from BME groups in health and social care research
2. Identify any reported factors that may promote or inhibit BME involvement in health and social care research.

## 2 METHODS

A systematic review was conducted and reported in accordance with the Preferred Reporting Items for Systematic Reviews and Meta‐Analyses (PRISMA) guidelines.^24^

### 2.1 Search strategy

A comprehensive search strategy included combinations of four main blocks of terms, including and relating to public involvement (patient, public, consumer, citizen, carer, lay, service users, stakeholder, family, relative, survivor), the type of involvement (participation, collaboration, engagement, partnership, consultation, user‐led, consumer or patient panel, advisory board/group/panel), health and social care research (health services, health care, public health, social care, etc.) and ethnicity^4^ (ethnic*, race, cultur*, minorit*) using a combination of MeSH terms wherever relevant and possible (see supplementary file S1 for example search strategy). Six main electronic bibliographic databases were searched for potential studies from January 1990 to November 2015 (and then updated in April 2016 to include any recent and relevant studies as the initial searches were >6 months old): MEDLINE, EMBASE, PsycINFO, Evidence Based Medicine (EBM) reviews (Cochrane library), Cumulative Index to Nursing and Allied Health Literature (CINAHL) and Healthcare Management Information Consortium (HMIC) were searched. Additionally, citation searches and reference lists of included studies and systematic reviews supplemented the database searches (see Figure 2).

A lack of medical subject headings (MeSH) for PPI has been previously reported, and MeSH terms on PPI need to be developed in order to identify more sensitive ways of searching.^10^ In order to compensate for this, and identify studies that were not mapped to MeSH terms, free‐text terms were used resulting in long and complex search string similar to Brett et al.^10^ This approach was necessary because there is no consistency in the way databases index studies relating to PPI, definitions, conceptualizations and poses challenges for developing search strategies.^10^ Moreover, several scoping exercises in different electronic databases were applied to maximize the sensitivity and specificity of the developed search strategy. For example, the search term “critical friend”^5^ did not yield any results; the term “PPI” was excluded as it minimized the retrieval of relevant studies as PPI has different meanings.

While the purpose of the review was to identify relevant articles focusing on BME‐PPI, studies utilizing CBPR and related participatory approaches identified by the searches and which met the inclusion criteria *were* included in this review (despite it not being used as a search term or being a focus) due to the fundamental shared characteristic of a philosophy of partnership and collaboration in both CBPR and PPI, between those conducting the research and those for whom the research is focused on or about. Furthermore, the use of INVOLVE's definition in this review, that is to look for research “carried out ‘with’ or ‘by’ members of the public” also meant there was scope to include any studies retrieved by the search which appeared relevant to the overall aims of the study. The overlap in terminologies used to describe “involvement” is a complex issue, and a detailed discussion comparing CBPR and PPI is outside the scope of this review. However, a table has been presented (see Appendix 1) to briefly offer an overview of similarities and differences between these two approaches.

### 2.2 Inclusion and exclusion criteria

Studies were included in this review if they met the following criteria:

*Population:* Adults (18 years or above) classified as being from a BME group(s) explicitly by the authors of the study within the paper itself. Members of *any* BME groups as defined by the authors of the studies themselves and from any country were included. Studies focusing on migrants including refugees, asylum seekers of different nationalities identified by authors as minority ethnic groups, are included even if detailed descriptors of their ethnicities were not available. In these cases, the population was defined on the basis of “countries of origin.” While the populations identified in this review as BME may be different (eg, indigenous peoples) due to characteristics such as language, ethnicity, culture, migration, all of these groups share similar key characteristics in that they are all likely to experience health inequalities, discrimination, racism and stigmatisation that can marginalize these populations and therefore are included in this review. If studies exclusively focused on majority groups or a combination of minority ethnic and majority groups where the data from minority ethnic groups was not clearly identifiable, then they were excluded.

*Types of studies:* All study designs reporting empirical, primary health or social care research regarding PPI of the population of interest as outlined above were eligible for inclusion.

INVOLVE's definitions of “the public” and “public involvement” in research were utilized in this review. INVOLVE defines the public involved in health and social care research as: “patients and potential patients; people who use health and social services; informal carers; parents/guardians; disabled people; members of the public who are potential recipients of health promotion programmes, public health programmes and social service interventions; and organisations that represent people who use services”. (p. 6)^1^

INVOLVE is a UK organization; however, the definitions are broad, encapsulate the concepts of interest and therefore allow for the capture of any relevant studies of interest. Furthermore, it has been previously utilized in reviews exploring the impact of PPI in health and social care research.^10^, ^26^

Finally, in order to be eligible for inclusion, studies were published between 1990 and 2016; 1990 was chosen as a starting point to capture any relevant publications leading up to the establishment of INVOLVE in 1996.

*Settings/Context:* Studies conducted in a primary or secondary health‐care setting or at the interface of such settings and/or social care research context.

### 2.3 Exclusion criteria

- PPI in service development and clinical audit
- Editorials, letters, commentaries, opinion pieces, theses and reviews, although the latter was used to identify other relevant studies for inclusion
- Studies discussing the role of people from ethnic minority backgrounds as research participants
- Studies not published in English
- Grey literature

### 2.4 Study selection

An EndNote library was utilized to combine and export the results of the searches from different databases. Duplicates (n=1804) were removed prior to the selection of studies (see Figure 2). Study selection was completed in two stages. First, two reviewers (SD and SC) independently screened titles and abstracts in order to identify eligible and relevant studies. Inter‐rater reliability was high (kappa coefficient=.89). Subsequently, full text of relevant studies was screened and reviewed in full by the first author for eligibility and a random subset sample of 50% were screened independently by a second reviewer (SC) (kappa coefficient=.73). Any disagreements were resolved through discussions.

### 2.5 Data extraction and critical assessment

**Figure 1:**
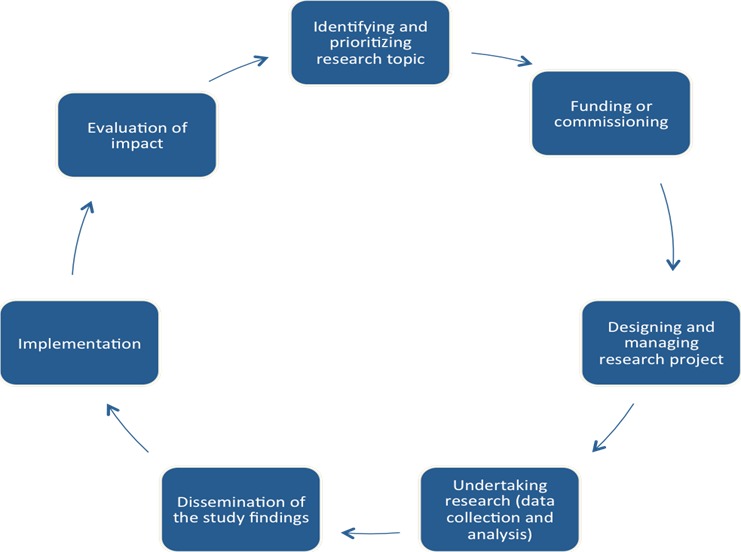
Research cycle adapted from INVOLVE website^27^, ^28^

A data extraction form was devised in Microsoft Excel and piloted on four randomly selected studies. The following descriptive data for included studies were extracted:

- *Study characteristics*—authors, year, country, type of study/study design, health topic focus
- *Participant characteristics*—types of people involved in health research (including ethnicity)
- *Main outcomes*—reported methods of recruitment^6^ and communication,^7^ reported levels of PPI (ie, consultation, collaboration or user control), description of PPI activities within the research study (eg, identifying and prioritizing research topics, research design and management, data collection, data analysis or dissemination) that is where they were involved in the research cycle^27^, ^28^ (see Figure 1), and, any reported factors to facilitate or inhibit BME involvement.

The first author extracted the data, and the second reviewer (SC) extracted a random sample of 20% (n=10) independently. No substantial disagreements were observed.

### 2.6 PPI involvement

Five PPI contributors were involved in reviewing the systematic review protocol, with three of the five PPI contributors coming from BME backgrounds. Three of the five PPI contributors had considerable experience of PPI in research. Two contributors who lacked prior research experience were provided with an informal educational session on systematic reviews, using a lay summary adapted from previous work.^29^ This education covered what a systematic review is, the processes involved and advantages and disadvantages of a systematic review. The purpose of this session was to provide them with a basic understanding of the systematic review process, to help them when reviewing the protocol. Despite this educational session, the PPI contributors felt that they did not have the relevant training and skills and as they were relatively new to research. Due to the availability of limited resources and time, it was agreed that the PPI contributors would not be involved further in the systematic review process.

PPI feedback from the review protocol was therefore more focused on clarifying and understanding the review process. However, the remaining three PPI contributors provided feedback on the review protocol, resulting in the addition of two new search terms (steering group and patient advocate). The PPI contributors also felt that there was a need to consider how minority ethnic groups were defined, due to the variety of possible definitions internationally. It was agreed that author definition would be an acceptable and consistent definition.

### 2.7 Critical assessment of the studies

There are difficulties when assessing the quality of PPI and related activities, as PPI activities vary from study to study and PPI activities within studies can be designed differently to the actual study itself.^10^ Therefore, this review assessed the quality of PPI‐related activities rather than appraising the quality of studies themselves, as it allowed a focus on the capture of PPI activity, which is the main outcome of interest for this review.

A critical appraisal checklist designed by Wright et al.^30^ was utilized to evaluate the quality of PPI reporting within published research. The first author extracted data and then a second reviewer (SC) checked a random sample of 20% independently. At this stage, disagreements were resolved through discussion with two other authors (SG and RM).

## 3 DATA SYNTHESIS

Data were reported in a narrative fashion due to the heterogeneous nature of the included literature.^31^ This entailed familiarization with the papers and identification of data pertaining to themes related to study aims/outcomes. Data were extracted in accordance with the relevant outcomes and have been summarized in descriptive form (See Tables 1, 2, 3) in order to draw conclusions about the available evidence.

**Table 1:** Characteristics of included studies

Author and Year	Country	Design	Health topic focus	Ethnicity
Allen et al. (2006)	USA	Mixed methods^a^	Alcohol abuse, sobriety	Alaska Native
Ameling et al. (2014)	USA	Quantitative	Hypertension	African Americans
Anderson‐Lewis et al. (2012)	USA	Mixed methods^a^	Hypertension	African American
Burrus et al. (1998)	USA	Quantitative	Diabetes	Black Americans
Chadiha et al. (2011)	USA	Quantitative	Health education and promotion	Older African Americans
Chen et al. (1997)	USA	Quantitative	Breast and cervical cancer	Korean American
Chesla et al. (2013)	USA	Quantitative	Type 2 diabetes	Chinese American
Choudhry et al. (2002)	Canada	Qualitative	Health promotion	South Asian immigrant women
Christopher et al. (2011)	USA	Multiple‐case study	Reduce health disparities	Native American
Dickson et al. (2001)	Canada	Qualitative	Health promotion	Aboriginal women
Dong et al. (2011)	USA	Qualitative	Elder mistreatment	Chinese
Fitzgerald et al. (2015)	Canada	Quantitative	Smoking cessation	Chinese
Gauld et al. (2011)	Australia	Qualitative	Brain injury	Aboriginal
Gibson et al. (2005)	Canada	Qualitative‐multimethod	Tuberculosis	Aboriginal
Gittlesohn et al. (2010)	Canada	Mixed methods^a^	Chronic disease prevention	Inuit
Gregg et al. (2010)	USA	Qualitative	Cervical cancer	Latino
Hayley et al. (2014)	USA	Qualitative	Eating, physical activity and sleeping behaviours	Burmese Refugee
Hull et al. (2010)	USA	Quantitative	Cancer	Hispanic
Isler et al. (2014)	USA	Curriculum development	HIV	Blacks
Ivey et al. (2004)	USA	Mixed methods^a^	CHD	Asian Indians
Johnson et al. (2009)	USA	Mixed methods^a^	Reproductive health care	Somali
Jones et al. (2010)	USA	Curriculum development	Pre‐term birth	African Americans
Knifton et al. (2012)	Scotland, UK	Qualitative	Mental Health	SA (Pakistani, Indian, Chinese titled MEG)
Larkey et al. (2009)	USA	Quantitative	Cancer prevention screening curriculum	Latino
Ma et al. (2012)	USA	Quantitative	Hepatitis B	Korean Americans
Ma et al. (2015)	USA	Quantitative	Cervical cancer	Vietnamese Americans
Maar et al. (2009)	Canada	Qualitative	Mental Health	Aboriginal
Matsunaga et al. (1996)	USA	Quantitative	Breast and cervical cancer	Native Hawaiian
McMullin et al. (2010)	USA	Not Stated	Diet, obesity, psychosocial factors related to food and nutrition for cancer prevention	Native Hawaiian
McQuiston et al. (2005)	USA	Grant writing	HIV	Latino
Mosavel et al. (2010)	USA	Evaluation	Cervical cancer	African American
Mott and Crawford (2008)	USA	Quantitative	HIV	African American
Newman et al. (2014)	USA	Evaluation	Diabetes	Zuni Indians
Nicolaidis et al. (2010)	USA	Qualitative	Depression	African American
Quinn (2014)	UK	Qualitative	Mental health	Asylum seekers and refugees
Rhodes et al. (2006)	USA	Intervention	HIV and STD	Latino men
Savage et al. (2006)	USA	Qualitative	Pregnancy and infant care	African American
Schultz et al. (2009)	USA	Case study	Cardiovascular disease and diabetes	African American and Hispanic
Shirazi et al. (2015)	USA	Qualitative	Breast cancer	Afghan
Springfield et al. (2015)	USA	Quantitative	Obesity among women (weight loss intervention)	African American
Street et al. (2007)	Australia	Qualitative	General health	Aboriginal
Voyle et al. (1999)	NewZealand	Evaluation	Health promotion	Indigenous
Vukic et al. (2009)	Canada	Qualitative	Mental health	First Nation
Wang et al. (2012)	USA	Mixed methods^a^	Diabetes	Chinese American
Watson et al. (2001)	USA	Evaluation	Oral health	Latino

a Mixed methods refer to studies utilizing quantitative and qualitative methods.

**Table 2:** Who gets involved and stages of involvement

Study ID	Who is involved?	How are they involved?	Identifying research agenda	Proposal/funding	Design	Development of tools (eg, questionnaires ads, info sheet consent)	Recruitment	Data collection	Analysis & interpretation	Dissemination
Allen et al. (2006)	Alaska native cultural groups with different work experience students	Co‐researcher involvement	X	X	Yes	X	Yes	Yes	Yes	X
Ameling et al. (2014)	Community members, local political leaders, HCPs, administrators, patients, insurers, representatives from city and state health departments, faith community reps and community organization leaders	Community Advisory Board	X	Yes	Yes	X	X	Yes	X	Yes
Anderson‐Lewis et al. (2012)	Members representing community‐based and civic organizations, city government and local health‐care agencies	Community Advisory Board	X	X	X	Yes	X	Yes	Yes	X
Burrus et al. (1998)	Local leadership organizations (such as representatives of the local black ministerial association), general and black medical associations, the health department, the county parks and recreation department, the media and those organizations with a clear stake in diabetes care (eg, the American Diabetes Association	Community Advisory Board	X	Yes	Yes	Yes	X	Yes	X	Yes
Chadiha et al. (2011)	Older urban African Americans (community residents, professionals and members of service organizations)	Community Advisory Board	X	X	X	X	Yes	Yes	X	X
Chen et al. (1997)	Korean American immigrants including community leaders	Community Advisory Board	X	X	Yes	Yes	Yes	X	Yes	Yes
Chesla et al. (2013)	Community organizations and members representing Chinese immigrants with type 2 diabetes, social and health service providers	Community Advisory Board and CBPR workgroup	X	X	Yes	X	Yes	X	X	X
Choudhry et al. (2002)	Women from Punjabi and Gujarati communities	Not reported	X	X	Yes	X	X	X	X	X
Christopher et al. (2011)	Various tribal members	Community Advisory Boards	X	Yes	Yes	Yes	Yes	X	X	Yes
Dickson et al. (2001)	Older Aboriginal women (grandmothers) and project advisory committee	Co‐researcher involvement	X	Yes	Yes	Yes	X	Yes	Yes	X
Dong et al. (2011)	Chinatown stakeholders and leaders through civic, health, social and advocacy groups, community centres, community physician and residents	Community Advisory Board	X	X	X	Yes	Yes	Yes	X	X
Fitzgerald et al. (2015)	Members of Mandarin and Cantonese communities	Key informants	X	X	Yes	Yes	Yes	X	X	Yes
Gauld et al. (2011)	Members from a range of Aboriginal, disability, health and academic organizations, and people external to both of these communities	Expert Advisory group	X	X	Yes	Yes	X	X	X	X
Gibson et al. (2005)	Members from different organizations with a view to ethnicity, networking experience, leadership skills and knowledge of community health	Community Advisory Committee	X	Yes	Yes	X	Yes	X	X	X
Gittlesohn et al. (2010)	Store staff, local health staff, community leaders, community members	Not reported	X	X	X	Yes	X	X	X	X
Gregg et al. (2010)	Local community leaders, community organiser, country and community health workers and a stay‐at‐home mother	Community Advisory Board	X	Yes	Yes	Yes	X	X	Yes	X
Hayley et al. (2014)	Local non‐profit organization serving Burmese refugees—community advisory representatives from four ethnic groups from Burma—Karen, Karenni, Kachin and Chin	Advisory group	X	X	Yes	X	Yes	Yes	X	X
Hull et al. (2010)	Community centre, members of the organization (Hispanics)	Not reported	X	X	X	Yes	Yes	Yes	Yes	Yes
Isler et al. (2014)	Community members, local political leaders, HCPs, administrators, patients, insurers, representatives from city and state health departments, faith community representatives and community organization leaders	Not reported	X	X	Yes	Yes	X	Yes	X	X
Ivey et al. (2004)	Organizations with members who were South Asians or had ties to South Asian communities. Individuals—Indian professionals, lawyers, physicians, other Asian Indian leaders	Advisor board	X	X	Yes	Yes	Yes	Yes	X	X
Johnson et al. (2009)	Health professionals, representatives from community‐based organizations, refugee resettlement agencies and immigration law experts	Community Advisory Board	X	X	Yes	Yes	X	Yes	X	X
Jones et al. (2010)	Community stakeholders, academics, researchers and government agencies	Community members	X	Yes	X	X	X	X	X	X
Knifton et al. (2012)	Mental health agencies, national antistigma campaign team and community groups representing three largest black and minority ethnic groups	Community coalition	X	X	Yes	Yes	Yes	Yes	X	X
Larkey et al. (2009)	Local professionals, lay community members and Juntos staff converged. Representatives (especially those of Latino background) from various community‐based and health organizations, including staff from clinics serving low‐income Hispanics; local project groups contracted to conduct tobacco cessation programmes; physicians from the Arizona Latino Medical Association; members from participating churches; and public school personnel	Hispanic Advisory board	X	Yes	Yes	Yes	Yes	Yes	X	X
Ma et al. (2012)	Community‐based organizations‐churches, two health‐care providers, academic institution	Community Advisory Board	X	X	Yes	Yes	Yes	X	X	X
Ma et al. (2015)	Vietnamese community leaders	Coalition	X	X	Yes	Yes	Yes	X	X	X
Maar et al. (2009)	Aboriginal elders, community members and local decision makers	Steering committee	X	X	Yes	X	X	X	Yes	X
Matsunaga et al. (1996)	Community representatives, health professionals and researchers	Steering committee	X	Yes	Yes	X	Yes	X	X	X
McMullin et al. (2010)	Community leaders and community partners	Collaborative	X	Yes	Yes	X	X	X	X	X
McQuiston et al. (2005)	Community members from Latino advocacy organizations	Not reported	X	Yes	Yes	X	X	X	X	X
Mosavel et al. (2010)	Mother‐daughter	Collaborative	X	X	X	X	X	X	Yes	X
Mott and Crawford (2008)	Stakeholders and representatives of the community under study, including persons living with HIV (consumers), advocacy groups, spiritual leaders recruited from black churches, political leaders, health‐care providers and various CBOs (agencies providing services to persons living with AIDS, social services)	Community Advisory Boards	X	Yes	Yes	Yes	Yes	X	X	X
Newman et al. (2014)	Community health representatives‐Zuni Indians	Not reported	X	X	X	Yes	X	Yes	X	X
Nicolaidis et al. (2010)	Not reported	Community partners	X	X	Yes	Yes	X	Yes	Yes	X
Quinn (2014)	Members of asylum seeker and refugee communities and leaders	Not reported	X	X	Yes	X	Yes	Yes	X	Yes
Rhodes et al. (2006)	Members of communities in action, representatives from local health and Latino serving community‐based organizations, religious organizations	Not reported	X	X	Yes	Yes	Yes	Yes	Yes	X
Savage et al. (2006)	Women who lived in the community	Community partners	X	Yes	Yes	Yes	Yes	X	Yes	X
Schultz et al. (2009)	Representatives from five health centres, neighbourhood organizations	Coalition	X	X	Yes	Yes	X	X	X	Yes
Shirazi et al. (2015)	Community leaders, health‐care providers, academic research partners, a cultural consultant, community navigators and women from the community	Community Advisory Board	X	X	Yes	Yes	X	X	X	X
Springfield et al. (2015)	Local leaders, health‐care providers, community members, advocates and local researchers who are experienced in CBPR	Community Advisory Board	X	X	Yes	Yes	Yes	X	X	X
Street et al. (2007)	Two Aboriginal community‐controlled health services, two government departments and eight universities and extensive networks into the Aboriginal health sector	Steering group	X	X	Yes	X	X	X	X	X
Voyle et al. (1999)	Principal of the employment training programmes operating at the marae; her human resources manager; SADP's Maori liaison worker, the diabetes specialist; the evaluator and a young female member of the marae	Partnership	X	X	Yes	Yes	X	X	X	X
Vukic et al. (2009)	Health directors of the 13 Mi'kmaq communities in Nova Scotia	Not reported	X	Yes	Yes	Yes	X	X	X	Yes
Wang et al. (2012)	Staff and senior clients from day care centres	Community Advisory Board	X	X	Yes	Yes	Yes	X	X	Yes
Watson et al. (2001)	Representatives of all community‐based organizations, as well as other individuals with diverse backgrounds, such as community lay people, health educators, social workers, administrators and local private dentists	Steering committee	Yes	X	Yes	Yes	X	X	X	X

**Table 3:** Reported inhibitors and facilitators to black and minority ethnic (BME) involvement

Author and Year	Inhibitors	Facilitators
Allen et al. (2006)	Not reported	Not reported
Ameling et al. (2014)	Not reported	Not reported
Anderson‐Lewis et al. (2012)	Not reported	Not reported
Burrus et al. (1998)	Not reported	Education on diabetes. Time was allocated for listening and discussing Community Advisory Board's perceptions of diabetes
Chadiha et al. (2011)	Not reported	Not reported
Chen et al. (1997)	Not reported	Not reported
Chesla et al. (2013)	Cultural challenges—finding ways to appropriately engage agency hierarchies, mix work and social time and negotiate protected time for community staff with limited prior research engagement	Face‐to‐face meetings with open agendas aided group members to voice concerns and explore culturally appropriate solutions.
Choudhry et al. (2002)	Lack of previous experience made them feel reluctant to take responsibility for certain components of the research process	Not reported
Christopher et al. (2011)	Not reported	Not reported
Dickson et al. (2001)	Not reported	Not reported
Dong et al. (2011)	Not reported	Not reported
Fitzgerald et al. (2015)	Not reported	Not reported
Gauld et al. (2011)	Not reported	Not reported
Gibson et al. (2005)	Not reported	Not reported
Gittlesohn et al. (2010)	Not reported	Not reported
Gregg et al. (2010)	Not reported	Not reported
Hayley et al. (2014)	Not reported	Not reported
Hull et al. (2010)	Not reported	Not reported
Isler et al. (2014)	Concerns with the level of expertise needed to contribute to the research process and understanding how their involvement would build on their skill set. Power differences, challenges with maintaining trust among members and extent to which individuals felt comfortable to speak in front of groups.	Not reported
Ivey et al. (2004)	Not reported	Not reported
Johnson et al. (2009)	Distrust	Not reported
Jones et al. (2010)	Not reported	Not reported
Knifton et al. (2012)	Not reported	Not reported
Larkey et al. (2009)	Not reported	Not reported
Ma et al. (2012)	Time constraint	Working closely with the pastors to make social and health concerns part of their mission. This helped gain their “buy in” to the programme as part of their overall pastoral goals. Efforts were made to increase trust and garner commitments from one another.
Ma et al. (2015)	Not reported	Not reported
Maar et al. (2009)	Not reported	Not reported
Matsunaga et al. (1996)	Conflicts because of historical distrust and difference in perspectives and priorities	Resolved conflicts through discussions and consensus and built trust gradually
McMullin et al. (2010)	Not reported	Not reported
McQuiston et al. (2005)	Not reported	Not reported
Mosavel et al. (2010)	Time commitment	Not reported
Mott and Crawford (2008)	Not reported	Compensation served as a form of recognition and contribution
Newman et al. (2014)	Not reported	Not reported
Nicolaidis et al. (2010)	Not reported	Not reported
Quinn (2014)	Not reported	Not reported
Rhodes et al. (2006)	Not reported	Not reported
Savage et al. (2006)	Not reported	Not reported
Schultz et al. (2009)	Not reported	Not reported
Shirazi et al. (2015)	Not reported	Not reported
Springfield et al. (2015)	Not reported	Not reported
Street et al. (2007)	Not reported	Not reported
Voyle et al. (1999)	Not reported	Not reported
Vukic et al. (2009)	Not reported	Not reported
Wang et al. (2012)	Awareness of distrust, inadequate communication, disregard of cultural beliefs and language	Researchers spent more time with community members to understand their problems and concerns as they may not have been researchers’ area of expertise. Use of bilingual researchers to overcome cultural and language barriers.
Watson et al. (2001)	Friction within community‐based organizations as a result of budget cuts prompting gaps in communication and collaboration. Different priorities of the communities	Not reported

### 3.1 Search results

**Figure 2:**
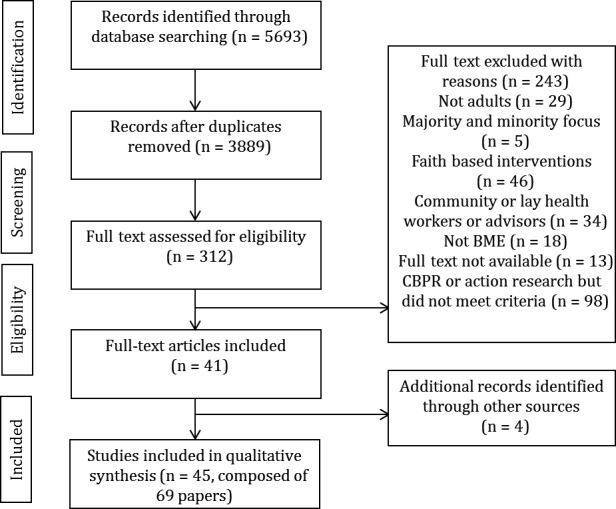
PRISMA flow diagram of study selection

In total, the number of titles and abstracts identified from searching the electronic databases were 5693 papers, and they were screened for eligibility. After reviewing titles and abstracts, this was reduced to 312 papers which were initially eligible for inclusion in the review. After reading full‐text articles, a total of n=41 individual studies (reported across 65 papers) were included. From citation searches and searching the reference lists, a further four studies were identified and included in this review (see Figure 2).

### 3.2 Characteristics of studies and populations

Table 1 presents an overview of the included studies. Tables 1 and 2 offer an initial description of the included studies and give an overview of who got involved, how they are involved and reported PPI activities during different stages of the research cycle were extracted. This allowed exploring, identifying and describing any patterns both across and within studies by tabulating the extracted data into different clusters as described in Tables 1 and 2.

### 3.3 Summary overview

#### 3.3.1 Populations and settings

Eleven study populations focused on African Americans,^32^, ^33^, ^34^, ^35^, ^36^, ^37^, ^38^, ^39^, ^40^, ^41^, ^42^ five studies on Aboriginal groups,^43^, ^44^, ^45^, ^46^, ^47^ four on Chinese,^48^, ^49^, ^50^, ^51^ three on South Asians,^52^, ^53^, ^54^ two on Native Hawaiian,^55^, ^56^ five on Latinos,^57^, ^58^, ^59^, ^60^, ^61^ two on Korean Americans,^62^, ^63^ one each on Zuni Indians,^64^ Somalis,^65^ Afghan,^66^ Native Americans,^67^ Alaska Natives,^68^ Inuit,^69^ indigenous groups,^70^ First Nations,^71^ Hispanics,^72^ Vietnamese Americans,^73^ various groups (Somalian, Eritrean, Pakistani, Iranian, Iraqi, Chinese and Sri Lankan),^74^ Burmese^75^ and African Americans and Hispanics.^76^ Thirty‐three studies were undertaken in the USA,^32^, ^33^, ^34^, ^35^, ^36^, ^37^, ^38^, ^39^, ^40^, ^41^, ^42^, ^48^, ^49^, ^50^, ^53^, ^55^, ^56^, ^57^, ^58^, ^59^, ^60^, ^61^, ^62^, ^63^, ^64^, ^65^, ^66^, ^67^, ^68^, ^72^, ^73^, ^75^, ^76^ seven in Canada,^43^, ^44^, ^46^, ^51^, ^52^, ^69^, ^71^ two each in UK^54^, ^74^ and Australia^45^, ^47^ and one in New Zealand.^70^

#### 3.3.2 Study types

Six studies employed a mixed‐methods design with both quantitative and qualitative elements,^39^, ^49^, ^53^, ^65^, ^68^, ^69^ and twelve studies included a quantitative design^32^, ^34^, ^38^, ^40^, ^42^, ^50^, ^51^, ^60^, ^62^, ^63^, ^72^, ^73^ with five randomized controlled trials,^34^, ^40^, ^42^, ^60^, ^73^ one pilot survey,^32^ repeated measures,^50^ cross‐sectional,^51^ quasi‐experimental,^63^ population‐based survey,^62^ intervention design^72^ and retrospective design.^38^ Fourteen studies utilized qualitative methods of data collection,^37^, ^43^, ^44^, ^45^, ^46^, ^47^, ^48^, ^52^, ^54^, ^61^, ^66^, ^71^, ^74^, ^75^ one was an ethnography^33^ and two utilized a case study design.^67^, ^76^ Furthermore, two studies described interventions,^55^, ^59^ one on grant writing,^58^ four on evaluation^36^, ^57^, ^64^, ^70^ and two on curriculum development.^35^, ^41^

#### 3.3.3 Study topics

A range of health conditions were identified: one study on alcohol abuse and sobriety,^68^ brain injury,^47^ chronic disease prevention,^69^ cardiovascular disease,^53^ cardiovascular disease and diabetes,^76^ depression,^37^ diet, obesity and psychological factors related to nutrition for cancer,^56^ obesity (weight loss),^42^ elder mistreatment,^48^ general health, eating, physical activity and sleep,^75^ general health,^45^ pregnancy and infant care,^33^ pre‐term birth,^35^ oral health,^57^ reducing health disparities,^67^ reproductive health,^65^ smoking cessation,^51^ tuberculosis,^44^ hepatitis B^63^ and cancer.^36^, ^55^, ^60^, ^61^, ^62^, ^66^, ^72^, ^73^ The remaining included diabetes,^32^, ^49^, ^50^, ^64^ health promotion,^38^, ^43^, ^52^, ^70^ HIV,^34^, ^41^, ^58^, ^59^ mental health^46^, ^54^, ^71^, ^74^ and hypertension.^39^, ^40^

#### 3.3.4 Involving PPI contributors

Studies generally did *not* report on *how* they identified the PPI contributors except for three studies wherein they employed snowballing interviews,^32^ through connections and networking with key community informants and community agencies.^33^, ^51^ Most of the studies (21)^32^, ^34^, ^38^, ^39^, ^40^, ^42^, ^43^, ^44^, ^47^, ^48^, ^49^, ^50^, ^53^, ^60^, ^61^, ^62^, ^63^, ^65^, ^66^, ^67^, ^75^ set up an advisory board as a means of involving different stakeholders. All studies except for one^40^ solely utilized face‐to‐face approaches (ie, meeting) as a mode of communicating with PPI contributors. One study^40^ utilized a combination of telephone, email and face‐to‐face approaches to communicate with PPI contributors in their research study. Studies utilized different techniques to establish partnership including: frequency of meetings (eg, monthly or biweekly), the nature and purpose (eg, update, training, workshop) of interactions that occur during the research process varied significantly and often no justification was provided as to why they chose to utilize particular techniques.

#### 3.3.5 Involvement in different stages of the research process

Forty‐three studies^32^, ^33^, ^34^, ^35^, ^36^, ^37^, ^38^, ^39^, ^40^, ^41^, ^42^, ^43^, ^44^, ^45^, ^46^, ^48^, ^49^, ^50^, ^51^, ^53^, ^54^, ^55^, ^56^, ^57^, ^58^, ^59^, ^60^, ^61^, ^62^, ^63^, ^64^, ^65^, ^66^, ^67^, ^68^, ^69^, ^70^, ^71^, ^72^, ^73^, ^74^, ^75^, ^76^ claimed that the projects were undertaken through partnering with different communities during the course of the research process; from Table 2 (columns 4‐11), it is clear that it did not necessarily translate in practice and the remaining two studies^47^, ^52^ utilized consultative forms of involvement. The notion of collaborative involvement where PPI contributors are *active* partners in the research process does not fully reflect in practice, as involvement activities took place at selective stages of the research process and never reportedly happened *throughout* the research process. For example, involvement most commonly took place during the design stage (35 of 45)^32^, ^33^, ^34^, ^37^, ^38^, ^39^, ^40^, ^41^, ^42^, ^43^, ^44^, ^45^, ^46^, ^47^, ^49^, ^50^, ^51^, ^52^, ^53^, ^54^, ^55^, ^57^, ^58^, ^59^, ^60^, ^61^, ^62^, ^65^, ^66^, ^67^, ^68^, ^70^, ^71^, ^73^, ^74^, ^75^, ^76^ (column 6), 29 studies on the development of various study materials^32^, ^33^, ^34^, ^37^, ^39^, ^41^, ^42^, ^43^, ^47^, ^48^, ^49^, ^51^, ^53^, ^54^, ^57^, ^59^, ^60^, ^61^, ^62^, ^64^, ^65^, ^66^, ^67^, ^69^, ^70^, ^71^, ^72^, ^73^, ^76^ (column 7) and 22 studies to enable recruitment of study participants^33^, ^34^, ^38^, ^42^, ^44^, ^48^, ^49^, ^50^, ^51^, ^53^, ^54^, ^55^, ^59^, ^60^, ^62^, ^67^, ^68^, ^72^, ^73^, ^74^, ^75^ (column 8), data collection (18)^32^, ^37^, ^38^, ^39^, ^40^, ^41^, ^43^, ^48^, ^53^, ^54^, ^59^, ^60^, ^64^, ^65^, ^68^, ^72^, ^74^, ^75^ (column 9) and data analysis and interpretation (11)^32^, ^33^, ^36^, ^37^, ^43^, ^46^, ^59^, ^61^, ^62^, ^68^, ^72^ (column 10). Involvement of PPI contributors was seldom during the preliminary stages of the research that is development of proposal/funding (13)^32^, ^33^, ^34^, ^35^, ^40^, ^43^, ^44^, ^55^, ^58^, ^60^, ^61^, ^67^, ^71^ (column 5), and only one study involved PPI contributors in identifying the research agenda^57^ (column 4). Only ten studies reported involving PPI contributors at the dissemination stage^32^, ^40^, ^49^, ^51^, ^62^, ^67^, ^71^, ^72^, ^74^, ^76^ (column 11).

#### 3.3.6 Reported inhibitors and facilitators to involvement

Studies often failed to report any inhibitors or facilitators to involving PPI contributors in their research with only 11 studies^32^, ^34^, ^36^, ^41^, ^49^, ^50^, ^52^, ^55^, ^57^, ^63^, ^65^ reporting some of the factors that facilitated or inhibited involvement (see Table 3). Inhibitors included cultural challenges,^50^ lack of previous experience and reluctance of PPI contributors to take responsibility,^52^ concerns about the level of expertise and lack of understanding of how their involvement can build their skill set,^41^ challenges in maintaining trust^41^ and distrust,^65^ conflicts because of distrust^55^ and difference in priorities,^55^, ^57^ time commitment,^36^ inadequate communication, disregarding cultural beliefs and language^49^ and friction as a result of budget cuts leading to gaps in communication.^57^ In contrast, factors such as compensation as a means of recognition and contribution,^34^ building trust and resolving conflicts gradually,^55^ efforts to spend more time with PPI contributors to understand their problems and concerns,^49^ use of bilingual researchers,^49^ working closely with PPI contributors, efforts to improve trust and garner commitments,^63^ open agendas to allow PPI contributors to voice concerns and explore culturally appropriate solutions,^50^ allocating time for listening and discussing health problems^32^ were all facilitators for involvement.

## 4 DISCUSSION

### 4.1 Summary of the findings

This is the first systematic review characterizing involvement, as opposed to participation, of BME groups in international health and social care research. The results presented in this review highlight where BME‐PPI activity occurs, who is involved and how they are involved as well as factors which inhibit or promote that involvement.

The majority of studies (forty of forty‐five) were from North America and focused on African Americans and/or Aboriginal/indigenous groups. Studies illustrated that researchers reported working primarily in partnership with the PPI contributors and PPI‐related activities mainly occurred at the design stage. CBPR and related participatory approaches were commonly utilized to involve BME contributors echoing the findings reported by Boote et al.^77^ There was poor reporting of the *extent* of involvement of PPI contributors and their contributions at the different stages of the research process as studies offered little information on them, making it difficult to extract sufficient information. For example, on several occasions, studies reported involvement in the planning and development stages without offering specific description of the different activities they were involved in (eg, development of proposal/funding (n=13), identifying research agenda (n=1), design stage (n=35), development of study materials (n=29), recruitment of study participants (n=22), data collection (n=18), data analysis and interpretation (n=11) and dissemination (n=10)) during each stage. No study offered any recommendations on the best practice of BME involvement or how BME involvement could be improved or performed differently. Some barriers (n=7) and facilitators (n=5) were reported, but this information was largely absent from the identified literature.

### 4.2 How the findings relate to the wider literature

The present review identified that there was minimal BME involvement during the latter stages of the research process, which reflects the findings of previous reviews exploring the impact of PPI^10^, ^77^, ^78^ and another which states that involvement occurred at the early stages of question identification or prioritization.^77^ With the exception of 11 studies,^32^, ^34^, ^36^, ^41^, ^49^, ^50^, ^52^, ^55^, ^57^, ^63^, ^65^ authors failed to report specific barriers and facilitators to BME involvement. Generally, studies did not offer any rationale for researchers’ decision to involve BME‐PPI contributors at certain stages of the research process over others, thus highlighting the gap between intended involvement versus actual involvement in practice, echoing the findings reported in previous reviews.^10^, ^77^, ^79^ A recent contribution to the literature by O'Reilly‐de Brún et al. 2016^80^ offered recommendations to meaningfully involve BME communities in *all* stages of research utilizing a participatory learning and action research approach and methodology. The authors recruited and trained migrants as Service User Peer Researchers who in turn helped recruit Migrant Service Users to the study. All methods were designed to be active, inclusive and collaborative with opportunities for meaningful involvement and engagement from research design to dissemination of study findings. Meaningful engagement for Service User Peer Researchers included training, capacity building, co‐design of documents, fieldwork, co‐analyse data and present Migrant Service Users’ views at conferences. For Migrant Service Users, meaningful engagement meant being actively, inclusively involved with shared responsibilities and undertook analysis in collaboration with Service User Peer Researchers. Peer researchers bridged links between migrant populations (often considered hard to reach) with academics allowing to create an active, sustained and productive community‐university partnerships.

The reported barriers, namely time constraints,^36^ lack of previous experience in research^41^, ^52^ or compensation can be generalized as issues experienced by *all* those who are usually involved in research.^17^ The review did not identify any studies offering insights into personal, social or cultural factors that were specifically relevant to people from BME groups that had an impact on their involvement.

Reviews exploring the impact of PPI^10^, ^11^ reflect on the intrinsic problem of assessing impact that is poor reporting and a lack of consistency, as there is a lack of clear structure when reporting PPI in peer‐reviewed journals. This makes it difficult to understand what works, for whom, under what circumstances and why.^10^, ^27^ Similarly, this review identified that evaluation of the quality of PPI activities by BME contributors within studies was challenging due to the variability in the way PPI activities were undertaken and lack of consistency in reporting PPI activities within studies. Studies also did not necessarily provide a comprehensive description of how they identified the PPI contributors and the extent to which they are involved in that particular context.

## 5 METHODOLOGICAL CRITIQUE

Rigorous search methods were designed to be comprehensive to ensure that all the existing published empirical evidence in this area was identified and the review was reported according to published guidelines. Despite national and international policy initiatives to promote PPI within health services research, to our knowledge, this is the first systematic review, specifically characterizing the international literature specifically on BME involvement in health and social care research. Specifically, who is involved, where involvement occurs and at what level as well as reported factors that appear to inhibit or facilitate BME involvement in such research.

Although, there is a wealth of evidence focusing on CBPR and related participatory approaches, hand‐searching of key journals for such studies were not undertaken as this was beyond the focus and scope of the current review. Relevant CPBR studies however were included if they were retrieved by our searches and met the inclusion criteria due to their fundamental shared characteristic of a philosophy of partnership and collaboration between those conducting the research and those for whom the research is focused on or about. The checklist developed by Wright et al.^30^ to assess the quality of PPI reporting was useful, but was limited to what was perceived and described as pragmatic involvement, with a focus on PPI‐related outcomes on research. Few studies reported barriers to PPI involvement and where they were reported, it was sometimes difficult to judge whether the reported barriers were from the perspectives of PPI contributors or study participants. In such cases, the first author (SD) read and re‐read the information and made a judgement to either include it as a PPI‐related barrier or exclude it as it referred to barriers to participation. Finally, it is also possible that substandard reporting of PPI could be a result of barriers such as limited word count in peer‐reviewed journals or because PPI reporting is not a priority making reviews such as this difficult to conduct.

Exclusion of grey literature was a limitation of this review; this is a common problem as factors such as access quality and heterogeneity of grey literature are stumbling blocks to its inclusion in a systematic review.^81^ However, as the current review focused solely on published academic peer‐reviewed literature in health and social care research and BME involvement within this context, the findings and lessons learnt are likely to be relevant for their intended audience of academics and policymakers.

## 6 IMPLICATIONS FOR PRACTICE AND FUTURE RESEARCH

Based on the findings of this review, there is a considerable gap in the extant health and social care literature regarding BME involvement, as outside of the USA, only a handful of health and social care studies appear to have included any BME involvement. Studies did not offer a rationale for involving BME contributors specifically and rarely discussed the added value (if any) of the PPI to the different stages of the research process. This draws attention to the need for a consistent form of reporting PPI activities in order to facilitate better quality assessment. The Guidance for Reporting Involvement of Patients and Public (GRIPP) checklist^82^ was developed and published in 2011 in order to improve consistency and enhance the quality of reporting of PPI activities within research studies. It offers a comprehensive list of issues that need consideration when reporting PPI activities; however, it does not offer information regarding how the PPI contributors were recruited or offer explicit consideration for BME involvement, any new iteration of the checklist should do so. Perhaps more critically, the GRIPP checklist does not appear to have been widely adopted within the health and social care literature as yet, even in studies published after 2011, none of the studies followed the guidance and journals seemingly do not routinely require authors to report in accordance with it. Thus, the literature fails to capture the range of PPI‐related activities due to lack of information or in‐depth description of PPI‐related activities. Furthermore, a key limitation of GRIPP checklist is that it was based on two systematic reviews and lacks international input.^82^

All studies included in this review did not utilize the term PPI; therefore, checklists that address PPI may not be used by the authors. Given that the empirical literature on BME involvement predominantly focuses on CBPR or participatory forms of PPI, future work should focus on developing and promoting adequate reporting tools to reflect this. Similarly, there is a need for development of common terminology, which can then be mapped in electronic databases. For example, a PPI‐specific MeSH term or filter such as the one developed by Rogers et al.^83^ (unavailable at the time this review was conducted) would be helpful when searching databases for relevant PPI‐related literature.

Importantly, the findings raise a number of issues that have relevance for health and social services policy, research and practice. Policy directives on PPI need to have an explicit consideration of the diversity of the PPI they are encouraging. While it is laudable to seek PPI involvement in health and social care research, policy needs to encourage the *right* type of involvement and that which reflects the population. In terms of the practice of PPI involvement, it is currently limited to particular stages. For example, although there is evidence suggesting that PPI can more generally lead to better dissemination and implementation of study findings by making it more accessible,^10^ only a minority (n=10) of included studies reported BME‐PPI in key stages such as dissemination.^32^, ^40^, ^49^, ^51^, ^62^, ^67^, ^71^, ^72^, ^74^, ^76^ Involvement during the dissemination phase can be significantly challenging for researchers, as the focus is on publishing results in peer‐reviewed journals, and place less emphasis on the implementation of findings.^10^ This might be especially difficult for researchers when involving people from BME groups, as they may not have the necessary language skills to be involved in producing traditional academic outputs such as conference and manuscripts. Therefore, there is a need to explore innovative ways of embedding BME involvement at this stage and broaden our view of dissemination more generally. PPI contributors’ language skills could be usefully employed to discuss the research findings more widely within the target population(s), as well as devising culturally appropriate dissemination strategies. PPI can be useful in developing the interpretation of data as it can offer different insights and identify aspects of research that may be of relevance to PPI contributors that is the intended users or recipients of that research.^10^ Despite this, the current review, as elsewhere (pg. 18), only identified a small number of studies (n=11) where BME‐PPI contributors were involved in data analysis and interpretation. While this may not always be possible, an indication of the consideration of this role for BME‐PPI contributors would be appropriate, particularly as interpretation of language and cultural meanings of research findings may otherwise be inaccurate. Practically, therefore, funders could seek funding applications which account for these difficulties that is those which seek additional finance to allow for language and culturally appropriate methods (eg, use of interpreters, translation of materials) of incorporating BME involvement at *all* stages of research when appropriate.

This review suggests that understanding specific factors, which may facilitate or inhibit BME involvement in health and social care research in specific BME populations, is also warranted. While this review grouped various ethnicities together in order to learn what may impact on BME‐PPI in general, it is important for health and social care studies in the future to target specific BME groups, where appropriate, as diversity exists between and within BME communities and different groups have different needs, priorities and issues. We have however attempted to uncover the common issues experienced by such groups and therefore any common learnings to be had prior to focusing on specific groups. Cultural issues were discussed broadly, and only one study^49^ discussed specific cultural issues in relation to language or how input from PPI contributors enabled inclusive participation as researchers had a better understanding of cultural issues. However, there is a paucity of information in relation to how other cultural factors could inhibit or facilitate involvement; for example, women from some BME groups might prefer to be involved as a part of a women's only group or involved individually.

Finally, and perhaps most importantly, we suggest that the manner in which PPI is being enacted by health and social care researchers is narrow,^16^ and the reinforcement of this approach by funders and journals only compounds this issue. One possible solution is to learn from the more established field of other participatory approaches. Power differentials between researchers and those researched are perhaps greater in PPI approaches than in other approaches which have co‐learning and shared decision‐making embedded. Adopting a more co‐produced, partnership approach to involvement will more likely enhance the probability of greater inclusivity, thereby helping to address the current imbalance of BME involvement in health and social care research. Beyond, this, agenda setting is also a key. A move towards initiatives such as the James Lind Alliance, for example, is important as they place the power of setting out a research agenda (within a health condition/setting) in terms of priority research questions from the point of view of those directly involved in health itself that is researchers and patients/public (and exclude researchers from this process). One could foresee specific BME populations being targeted and specific research questions being developed, and then translated into partnership working in order to answer those questions.

## 7 CONCLUSION

This review has identified the current state of the international empirical literature on BME involvement in health and social care research. Overall, the evidence base is considered to be weak as there is limited information on the nature and content of BME‐PPI‐related activities within health and social care studies and requires further substantive development in terms of understanding factors that influence BME involvement as opposed to involvement more generally, and how PPI can be made more inclusive.

## CONFLICT OF INTEREST

The authors declare no potential conflict of interests with respect to the research, authorship and/or publication of this article.

## Notes

1 By participation, we mean taking part in research either by completing questionnaires, taking part in interviews or focus groups.

2 By engagement, we mean sharing information about research with members of the public or disseminating study findings to research participants or members of the public.

3 For the purposes of this review, people involved in health and social care research will be referred to as PPI contributors.

4 Ethnicity‐related search terms were utilized from a previously published systematic review^25^

5 PPI contributors are sometimes referred to as critical friend.^25^^.^

6 The term “recruiting” here refers to the recruitment of PPI contributors to either carryout research (user‐controlled or as peer interviewers) or work in partnership with researchers rather than being participants/subjects in a research study. Methods of recruiting could include advertisements in GP practices, online, etc.

7 Methods of communication here include face‐to‐face, telephone, online via e‐mail or forum, postal.

## Appendix 1 Similarities and differences between PPI and CBPR

### 1.1

**Table:**

	PPI	CBPR
Definition	Most commonly used definition—INVOLVE define PPI as “research that is carried out ‘with’ members of the public or ‘by’ members of the public rather than'to’, ‘about’ or ‘for’ them” (p.1).^1^, ^77^	No consensus on an accepted definition of participatory health research. Viswanathan et al. (2004)^84^ define CBPR as *“a collaborative research approach that is designed to ensure and establish structures for participation by communities affected by the issue being studied, representatives of organizations, and researchers in all aspects of the research process to improve health and wellbeing through taking action, including social change.”* (p.22).
Approach and not a methodology	PPI is an approach that can be embedded into the research process at any stage.	CBPR is also an approach that can be incorporated into any stage of the research process.
Origins (top‐down/bottom‐up)	Within the UK, especially in the health policy or health service research context, PPI has taken a top‐down approach, either as part of official government policy imperative or as a requirement for evidence of PPI by the research funding programmes. There are different rationales for PPI which include moral (right thing to do), instrumental (involvement as a mechanism to achieve better aims) and substantive arguments tend to focus on the publics’ contribution towards quality of research^85^, ^86^ and typically the researcher controls and leads the projects.	CBPR focuses on reducing health disparities.^87^ Here, research is led and controlled by the community with little or no researcher involvement. However, CBPR can also be in the form of collaboration between researchers and community partner. It is a grassroots approach that aims to improve health of the community and eliminate disparities through joint ownership of the research project. Minkler and Wallerstein^87^ describe CBPR as a part of continuum with action research on one end to participatory research or participatory action research on the other end (more emancipatory approaches).
Individual vs Collective involvement	PPI tends to focus on individuals or small groups (involvement happens at individual level or collectively). PPI can include collaborative or partnership working, use of a advisory or steering committee as a means of involving patients and public in research. There is emphasis on partnership as the definition of PPI suggests working “*with”* members of the public.	CBPR focuses on collective identity that is community, and this is a reflection of who, how and where research takes place, emphasizing the importance of equitable partnership throughout the research process, relevant to community and is *community based* rather than merely *community placed*.^88^
Shared decision making and ownership	PPI can be about shared decision making and sharing ownership. However, the extent to which this happens depends on the a number of factors including needs of the project, the level of PPI utilized in the project (eg, consultation, collaboration/user controlled) and also the stages of involvement (ie, if the researcher chooses how and to what extent the PPI members are involved and whether or not the PPI members have a say in how they want to be involved). Shared decision making and ownership is *not* the central principle.	Viswanathan et al. (2004)^84^ emphasize that CBPR is (1) co‐learning about issues of concern and, within those, the issues that can be studied with CBPR methods and reciprocal transfer of expertise; (2) sharing of decision‐making power; and (3) mutual ownership of the products and processes of research. The end result is incorporating the knowledge gained with taking action or effecting social change to improve the health and well‐being of community members.^82^Shared decision making and ownership *is* a required principle^89^
Minority ethnic groups and other groups	Advocates of PPI suggest that it is challenging to involve people of BME groups. 10	Evidence seemingly implies that CBPR is the best way to meaningfully involve members of BME groups/vulnerable groups^80^, ^84^, ^87^, ^88^
Focus of Impact or outcomes	Impact of PPI can occur at project level, for researchers, for PPI members involved.^4^, ^12^, ^26^, ^27^	There is shared learning taking place. However, the end outcome is about social change or benefits for community that is capacity building.^84^, ^87^, ^88^ Here, both participation and the process are regarded as impact.^89^
