# Supplementary material for: Black and minority ethnic group involvement in health and social care research: A systematic review
Source: Health Expect. 2017 Aug 15;21(1):3–22. doi: 10.1111/hex.12597 (PMC5750731; doi:10.1111/hex.12597)
Supplement: Supplementary file 1 [file HEX-21-3-s001.pdf]

# S1: Full MEDLINE search strategy

**Searches**

| | |
|---|---|
| 1 | (ethnic or minorit*).ab,ti. |
| 2 | (BME or black ethnic minorit* or black minorit* ethnic*).mp. |
| 3 | asylum seeker*.ab,ti. |
| 4 | (migrant* or immigrant*).ab,ti. |
| 5 | (multicultural or multi-cultural).ab,ti. |
| 6 | (cross-cultural or crosscultural).ab,ti. |
| 7 | (trans-cultural or transcultural).ab,ti. |
| 8 | (multi-rac* or multirac*).ab,ti. |
| 9 | (multiethnic or multi-ethnic).ab,ti. |
| 10 | refugee*.ab,ti. |
| 11 | (multi-lingu* or multilingu*).ab,ti. |
| 12 | (ethno-cultur* or ethnocultur*).ab,ti. |
| 13 | (socio-cultural or sociocultural).ab,ti. |
| 14 | (divers* or diverse population* or cultural diversity).ab,ti. |
| 15 | (south asian* or bangladeshi* or pakistani* or indian* or sri lankan*).mp. |
| 16 | (asian* or east asian* or chinese or taiwanese or vietnamese or korean* or japanese).mp. |
| 17 | (afro-caribbean* or african-caribbean* or caribbean or african* or black* or afro*).mp. |
| 18 | (islam* or hindu* or Sikh* or buddhis* or muslim* or moslem* or christian* or catholic* or jew*).ab,ti. |
| 19 | ethnic group*.mp. |
| 20 | ((ethnic or linguistic) adj diversity).ab,ti. |
| 21 | (transient adj (group* or population*)).ab,ti. |
| 22 | acculturation.ab,ti. |
| 23 | (faith* or belief* or religion*).ab,ti. |

| | |
|---|---|
| 24 | ethnic*.ab,ti. or exp ethnic groups/ |
| 25 | minorit*.ab,ti. or exp minority groups/ |
| 26 | race.mp. or rac*.ab,ti. |
| 27 | cultur*.ab,ti. or exp Culture/ |
| 28 | exp continental population groups/ |
| 29 | aboriginal.mp. or aborigin*.ab,ti. |
| 30 | continental population groups.mp. or Continental Population Groups/ |
| 31 | 1 or 2 or 3 or 4 or 5 or 6 or 7 or 8 or 9 or 10 or 11 or 12 or 13 or 14 or 15 or 16 or 17 or 18 or 19 or 20 or 21 or 22 or 23 or 24 or 25 or 26 or 27 or 28 or 29 or 30 |
| 32 | consumer participation/ |
| 33 | patient participation/ |
| 34 | 32 or 33 |
| 35 | (patient* adj1 (participat* or involv* or engag* or partnership or partners or collaborat* or consult*)).ab,ti. |
| 36 | (public adj1 (participat* or involv* or engag* or partnership or partners or collaborat* or consult*)).ab,ti. |
| 37 | (user* adj1 (participat* or involv* or engag* or partnership or partners or collaborat* or consult*)).ab,ti. |
| 38 | (service user* adj1 (participat* or involv* or engag* or partnership or partners or collaborat* or consult*)).ab,ti. |
| 39 | (consumer* adj1 (participat* or involv* or engag* or partnership or partners or collaborat* or consult*)).ab,ti. |
| 40 | (lay adj1 (participat* or involv* or engag* or partnership or partners or collaborat* or consult*)).ab,ti. |
| 41 | (citizen* adj1 (participat* or involv* or engag* or partnership or partners or collaborat* or consult*)).ab,ti. |
| 42 | (carer* adj1 (participat* or involv* or engag* or partnership or partners or collaborat* or consult*)).ab,ti. |
| 43 | (caregiver* adj1 (participat* or involv* or engag* or partnership or partners or collaborat* or consult*)).ab,ti. |
| 44 | (customer* adj1 (participat* or involv* or engag* or partnership or partners or collaborat* or consult*)).ab,ti. |
| 45 | (client* adj1 (participat* or involv* or engag* or partnership or partners or collaborat* or |

| | |
|---|---|
| | consult*)).ab,ti. |
| 46 | (community* adj1 (participat* or involv* or engag* or partnership or partners or collaborat* or consult*)).ab,ti. |
| 47 | (stakeholder* adj1 (participat* or involv* or engag* or partnership or partners or collaborat* or consult*)).ab,ti. |
| 48 | ((patient* and public) adj1 (involv* or participat* or engag* or partnership or partners or collaborat* or consult*)).ab,ti. |
| 49 | (user led or user-led or lay control or user control).ab,ti. |
| 50 | ((representative* or patient representative* or patient advocate* or expert by experience or famil* or relative* or survivor*) adj1 (participat* or involv* or engag* or partnership or partners or collaborat* or consult*)).ab,ti. |
| 51 | ((patient* or consumer* or citizen* or advisory) adj1 board*).ab,ti. |
| 52 | ((patient* or consumer* or citizen* or advisory) adj1 group*).ab,ti. |
| 53 | ((patient* or consumer* or citizen* or advisory) adj1 panel*).ab,ti. |
| 54 | (citizen* adj1 (jury or juries)).ab,ti. |
| 55 | 35 or 36 or 37 or 38 or 39 or 40 or 41 or 42 or 43 or 44 or 45 or 46 or 47 or 48 or 49 or 50 or 51 or 52 or 53 or 54 |
| 56 | 34 or 55 |
| 57 | research.ab,ti. |
| 58 | health services research.mp. or exp health services research/ |
| 59 | social care research.mp. |
| 60 | social service*.mp. |
| 61 | public health.mp. or exp public health/ |
| 62 | psychology.mp. or exp psychology/ |
| 63 | psychiatry.mp. or exp psychiatry/ |
| 64 | sociology.mp. or exp sociology/ |
| 65 | nursing.mp. or exp nursing/ |
| 66 | (mental health or mental healthcare).mp. or exp mental health/ |
| 67 | secondary care.mp. or exp secondary care/ |
| 68 | tertiary care.mp. or exp tertiary healthcare/ |
| 69 | 58 or 59 or 60 or 61 or 62 or 63 or 64 or 65 or 66 or 67 or 68 |
| 70 | Family Practice/ |

| | |
|---|---|
| 71 | Primary Health Care/ |
| 72 | Physicians, Family/ |
| 73 | Community Health Services/ |
| 74 | Community Dentistry/ |
| 75 | Community Health Nursing/ |
| 76 | Community Mental Health Services/ |
| 77 | Community Pharmacy Services/ |
| 78 | Home Care Services/ |
| 79 | Community Mental Health Centers/ |
| 80 | family pract$.tw. |
| 81 | general practice$.tw. |
| 82 | community based.tw. |
| 83 | community care.tw. |
| 84 | family medicine.tw. |
| 85 | family physician$.tw. |
| 86 | primary care.tw. |
| 87 | (primary health care or primary healthcare).tw. |
| 88 | family doctor$.tw. |
| 89 | primary medical care.tw. |
| 90 | general physician$.tw. |
| 91 | general practitioner$.tw. |
| 92 | primary care practitioner$.tw. |
| 93 | (community adj (health or healthcare or health care)).tw. |
| 94 | primary healthcare team$.tw. |
| 95 | primary health care team$.tw. |
| 96 | primary medical care team$.tw. |
| 97 | practice nurse$.tw. |
| 98 | practice manager$.tw. |
| 99 | (gpsi or gpwsi).tw. |
| 100 | (practitioner$ adj3 special interest$).tw. |
| 101 | (primary care or primary health care or general practice or family practice or family |

| | |
|---|---|
| | medicine).nw. |
| 102 | 70 or 71 or 72 or 73 or 74 or 75 or 76 or 77 or 78 or 79 or 80 or 81 or 82 or 83 or 84 or 85 or 86 or 87 or 88 or 89 or 90 or 91 or 92 or 93 or 94 or 95 or 96 or 97 or 98 or 99 or 100 or 101 |
| 103 | 69 or 102 |
| 104 | 57 and 103 |
| 105 | 31 and 56 and 104 |
| 106 | limit 105 to (english language and humans and yr="1990 -Current") |
